# Relationship Between Legal Blindness and Depression

**Published:** 2019-10-01

**Authors:** Matías Osaba, Jimena Doro, Malena Liberal, Jennifer Lagunas, Irene C Kuo, Víctor E Reviglio

**Affiliations:** 1 Instituto de la Visión Cerro, Sanatorio Allende - Sede Cerro, Córdoba, Argentina; 2 Facultad de Medicina, Universidad Católica de Córdoba, Córdoba, Argentina; 3 Centro de Investigación y Desarrollo en Inmunología y Enfermedades Infecciosas, Consejo Nacional de Investigaciones Científicas y Técnicas, Córdoba, Argentina; 4 Eye Care Service, Hospital Córdoba, Córdoba, Argentina.; 5 Wilmer Eye Institute, Department of Ophthalmology, Johns Hopkins University School of Medicine, Baltimore, MD, USA

**Keywords:** Blindness, Depression, Visually Impaired Persons, Zung Scale, Barthel Index.

## Abstract

The higher prevalence rates of depression in visually-impaired individuals than the general population indicates that the condition per se increases the risk of depression. A person that is aware of the progressive loss of visual acuteness may have feelings of insecurity, anxiety, loss of independence and changes in social functioning, leading to depression. Several studies assessing the association between depressive symptoms and severity of vision loss have yielded inconsistent results. Some do not show any association, whereas others reported that depression severity is higher in those with substantial vision loss. The general aim of this manuscript was to determine the prevalence of depression in patients diagnosed with legal blindness in the Eye Care Service at the Hospital Córdoba between June 2016 and June 2017. The study sample consisted of 41 patients. The level of depression was assessed using the Zung scale and the degree of dependence in daily life activities was defined using the Barthel index. Data was anonymized for inclusion in a computer database and statistical confidentiality was protected. Data was analyzed using InfoStat statistical software. The results revealed a relation between legal blindness, degrees of dependency and depressive symptoms in patients of the Eye Care Service of the Hospital Córdoba. It is very important for health professionals to be trained to detect early signs and symptoms of depression and have the necessary tools for such an approach.

## INTRODUCTION 

The negative impact of vision loss on life quality, social interaction and psychological functioning has been well established [1, 2]. The higher prevalence rates of depression in visually-impaired individuals than the general population [3] indicates that the condition per se increases the risk of depression. A person that is aware of the progressive loss of visual acuteness may have feelings of insecurity, anxiety, loss of independence and changes in social functioning, leading to depression [4]. The number of patients with visual impairment has increased significantly over the years worldwide, reaching approximately 253 million in 2017, of whom 36 million are considered blind [5]. This phenomenon can be mainly attributed to the increase in life expectancy and the world population growth [6]. In Argentina, data obtained from the rapid assessment survey on avoidable blindness conducted in 2013 by the Ministry of Health [7] showed a prevalence of bilateral blindness of 0.7% (75648 individuals), and severe and moderate visual impairment of 2.6% and 9.1%, respectively (with greater incidence in women). In the province of Córdoba, the “*Anuario Estadístico Nacional en Discapacidad*” of 2016 recorded 798 people only with visual impairment, with a slight dominance of males (52.01%) [8]. On the other hand, world health organization (WHO) indicates that depression is a common illness that affects more than 300 million people, and is the leading cause of disability worldwide [9]; these facts make depression a global concern. 

Systematic reviews evidence that the prevalence of depressive symptoms in visually impaired individuals is variable, ranging between 14% and 44% [10-12]. Moreover, several studies assessing the association between depressive symptoms and severity of vision loss have yielded inconsistent results. Some do not show any association [13, 14], whereas others reported that depression severity is higher in those with substantial vision loss [15]. The Diagnostic and Statistical Manual of Mental Disorders, Fifth Edition (DSM) [16] classifies depression as slight, moderate and severe, based on the number, type and intensity of symptoms and the degree of functional impairment. Accordingly, it is important to differentiate the Visual Impairment and Legal Blindness (LB). In 1996, Arditi and Rosenthal proposed the definition of visual impairment as visual acuity lower than 3/10 in the better-seeing eye with the best correction and visual field (VF) below 20 degree. Whereas according to the WHO, LB refers to a patient with a VA of less than 1/10 in the better-seeing eye to light perception and/or a VF equal to or lower than 10 degree after a treatment and/or standard refraction correction. Use of scales that measure the Activity of Daily Living (ADL) has always been a matter of debate since the information they provide has been poorly studied [17]. Several ADL scales have been used; however, the Barthel scale is probably the best one and the most widely used in researches [18]. The Barthel scale has been validated in numerous studies, showing a low discrepancy between what patients state that they can do and what they really do [19]. The analysis of the items included in the Barthel scale supported its use in visually-impaired patients [20] since it measures the degree of independence of a person to perform daily activities. The Zung Self-Rating Depression Scale is a 20-item scale used by physicians in the primary care setting to identify depressive symptoms [21, 22]. This scale has been validated and analyzed in different languages [23] and in diverse specific populations, such as patients with cancer, people with cognitive alterations and Parkinson’s disease, university students and physicians [24]. The sensitivity of the scale was also determined to be suitable for detecting depression and as a continually used tool for research to measure clinical severity in patients [25]. The present work aimed to investigate the level of depression and degree of dependence in ADLs in patients with legal blindness.

## METHODS

A descriptive, observational, retrospective study was conducted using secondary source data. The medical records belonged to 41 consecutive patients who consulted spontaneously or referred to the Eye Care Service of the Hospital Córdoba, Córdoba, Argentina between June 2016 and May 2017. Patients 16 years of age or older with a diagnosis of legal blindness (VA equal to or lower than 1/10 -decimal notation- in the better eye with the best refractive correction and/or VF lower than 10 degree) [26] who responded the Zung and Barthel questionnaires were eligible. Patients using oral corticosteroids or antidepressants, those with thyroid disease and those with incomplete data were excluded [19]. The variables analyzed in this study were sex, age, level of depression correlated with patient’s score on the Zung scale and degree of dependence indicated by the Barthel questionnaire. Level of depression was assessed using the Zung Self-Rating Depression Scale [21, 27]. The degree of dependence for performing ADLs was defined using the Barthel index [28], a tool that measures the capacity of a person to perform daily activities and estimates the degree of independence. Those scales have been validated at the national and international levels [29-31] and used in several studies of similar characteristics. The Zung Self-Rating Depression Scale is a self-rating scale composed of 20 statements related to depression; half are worded positively and the half negatively. It is widely used as a screening tool, covering affective, psychological and somatic symptoms associated with depression. Somatic and cognitive symptoms have a high weight, with 8 items for each group; the scale is completed with two items related to the mood status and the other two to psychomotor symptoms. Each statement is assigned a value from 1 to 4 (a little of the time, some of the time, a good part of the time, most of the time). The scoring range is between 20 and 80, with 25-49 indicating a normal range, 50-59 mild depression, 60-69 depression and 70 severe depression. The Barthel index is a widely used tool to measure the capacity of an individual to perform 10 basic ADLs and provides a quantitative estimation of the subject’s level of dependency. Each activity is assigned a value of 0, 5, 10 or 15. The total range varies between 0 (completely dependent) and 100 (completely independent). Dependency is classified according to the scoring as independent (100), mild dependency (≥ 60), moderate dependency (59 to 45), severe dependency (44 to 20) and totally dependent (≤ 20). At first we obtained an ethical approval by the Education and Professional Training Committee of the Hospital Córdoba. All patients signed a written informed consent. Data was anonymized for the inclusion in a computer database and statistical confidentiality was protected. Data was analyzed using InfoStat statistical software (Facultad de Ciencias Agropecuarias, UNC, Argentina). The categorical variables were described according to their frequencies. The results of the measurable variables were expressed as mean ± standard error and comparison between groups (sexes) were made using an analysis of variance (ANOVA). Statistical significance was considered as P < 0.05. 

## RESULTS

**Figure 1:**
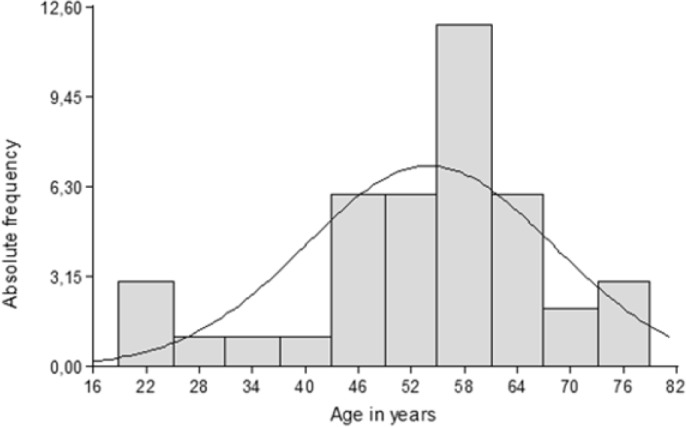
Age Distribution of the Study Population

**Figure 2:**
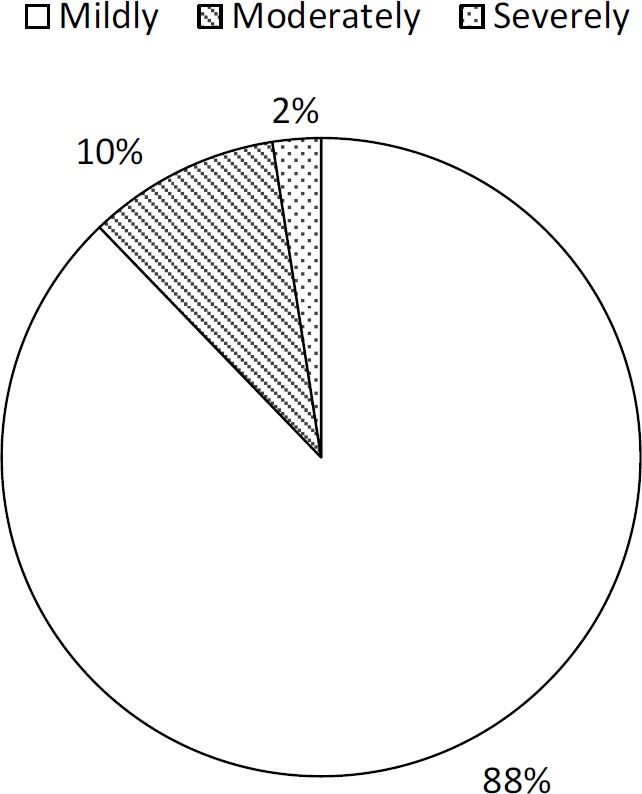
Results Obtained From the Barthel Scale for Degree of Dependence in Daily Life Activities of the Study Population. Note: Mildly: mildly dependent; moderately: moderately dependent; severely: severely dependent

The study sample consisted of 41 patients aged between 19 and 79 years (most patients aged 46 and 64 years), as shown in Figure 1. The participants were 66% males and 34% females. Despite the higher number of males, there were no statistically significant differences in the age of patients when comparing both sexes (P = 0.4033). According to the Zung scale, 29% of patients had depression, of whom 12% had mild depression, 5% moderate depression and 12% severe depression. The results of the Barthel scale showed in Figure 2 represent that 100% of the participants exhibited dependency for the execution of ADLs, with 88% being mildly dependent, 10% moderately dependent and 2% severely dependent. According to the results of the Barthel and Zung scales (Table 1), only 9.76% of patients exhibited mild dependency in daily activities and mild depression. However, 70.73 of our patients had mild dependency to perform activities of daily living according to the Barthel scale, and no depression according to the Zung scale. Of patients with moderate or severe degrees of dependency performing ADLs, 9.76% had Zung scores correlated with moderate to severe depression.

## DISCUSSION

The current study revealed that less than one-third of patients with legal blindness had depression. However, all of them exhibited some degree of dependency for the execution of ADLs, with the majority being mildly dependent, and only 12% had moderate to severe dependence. Few conclusive studies have investigated visual impairment and its effect on depression.

**Table 1:** Patients Characteristics According to Barthel and Zung Scores

Barthel Scale (Degree of dependence in daily life activities)	ZUNG Scale (Level of depression)	Total Number	Percentage (%)
Severe dependence	Severe depression	1	2.44
Mild dependence	Mild depression	4	9.76
Mild dependence	Moderate depression	1	2.44
Mild dependence	Normal	29	70.73
Mild dependence	Severe depression	2	4.88
Moderate dependence	Mild depression	1	2.44
Moderate dependence	Moderate depression	1	2.44
Moderate dependence	Severe depression	2	4.88
Total		41	100.00

Prevalence of depressive symptoms was found to vary between 14% and 44% in patients with visual impairment [10, 11] depending on the population characteristics and the metric used to assess depression or depressive symptoms. In our study, 29% of patients had Zung scores correlated with mild, moderate or severe depression. Vision is highly important for ADLs and enjoyment of life. Therefore, it is understandable that vision loss would have profound negative effects on mood. Although legal blindness is not a terminal disease, it can affect daily or social activities and quality of life [32], causing difficulties in ADLs, such as getting dressed, eating, writing, moving, communicating or interacting with others. Moreover, legal blindness can have a negative impact on social function and significantly reduce independence [33]. These facts may explain the feelings of distress, hopelessness and personal devaluation represented by questions on the Zung scale among individuals with blindness included in our study. The present study showed a relation between the degree of alteration of daily activities and the level of depression; 8.32% of patients with mild dependency for ADLs met symptom thresholds for moderate to severe depression.

The use of the Zung scale is an important contribution for research and screening mental illnesses or disorders such as depression. This fact is especially important since depressive disorders are underdiagnosed in primary care centers in developed countries. Therefore, the Zung scale is a reliable and no-cost tool for general health care professionals and specialists [34]. Most studies that investigated depression and blindness were conducted in samples of elderly patients and found an association between advanced age and prevalence of depression [10]. The results of the present study did not allow us to infer such association; indeed, the prevailing age of the sample was 46 and 64 years, since the population attending the Hospital Córdoba is generally is of heterogeneous age. The patients involved in this study are outpatients and with irreversible blindness, which is in contrast to the inclusion criteria in some investigations [13, 35, 36], including patients from a nursery home with correctable low vision. For the design of this work, the Mental Health Department of the Córdoba Hospital was consulted for advice on choosing the most appropriate surveys for this research work. Likewise, we used surveys that were currently used, that could be performed by any member of the health team and that was validated to be used in Spanish [37, 38].

The limitation of this work would be the number of patients. Recruiting patients who agree to participate in this research and meet the inclusion criteria is an arduous task. As strength, the work is one of the first of its kind at the provincial and national levels. Finally, it is suggested to perform further studies at a multicenter level in different health institutions with larger sample size in Argentina.

## CONCLUSIONS

There was a relation between legal blindness, degrees of dependency and depressive symptoms in patients of the Eye Care Service of the Hospital Córdoba.

Finally, interdisciplinary work is necessary involving eye care, diabetes, clinical medicine and mental health services to detect patients with legal blindness at risk of depression. It is very important for health professionals to be trained to detect early signs and symptoms of depression and have the necessary tools for such an approach. Interdisciplinary work and early diagnosis are fundamental tools to ensure support to patients and reduce the impact of consequences of having these problems. 

## DISCLOSURE

Ethical issues have been completely observed by the authors. All named authors meet the International Committee of Medical Journal Editors (ICMJE) criteria for authorship of this manuscript, take responsibility for the integrity of the work as a whole, and have given final approval for the version to be published. No conflict of interest has been presented.

## Funding/Support:

None.
